# Sex differences in the corpus callosum in preschool-aged children with autism spectrum disorder

**DOI:** 10.1186/s13229-015-0005-4

**Published:** 2015-05-13

**Authors:** Christine Wu Nordahl, Ana-Maria Iosif, Gregory S Young, Lee Michael Perry, Robert Dougherty, Aaron Lee, Deana Li, Michael H Buonocore, Tony Simon, Sally Rogers, Brian Wandell, David G Amaral

**Affiliations:** The MIND Institute, University of California at Davis, School of Medicine, 2805 50th Street, Sacramento, CA 95817 USA; Department of Psychiatry and Behavioral Sciences, School of Medicine, University of California at Davis, Sacramento, CA 95817 USA; Department of Public Health Sciences, School of Medicine, University of California at Davis, Davis, CA 95616 USA; Department of Psychology, Stanford University, Stanford, CA 94305 USA; Center for Cognitive and Neurobiological Imaging, Stanford University, Stanford, CA 94305 USA; Department of Radiology, School of Medicine, University of California at Davis, Sacramento, CA 95817 USA

**Keywords:** Connectivity, Diffusion tensor imaging, Longitudinal, MRI, White matter

## Abstract

**Background:**

Abnormalities in the corpus callosum have been reported in individuals with autism spectrum disorder (ASD), but few studies have evaluated young children. Sex differences in callosal organization and diffusion characteristics have also not been evaluated fully in ASD.

**Methods:**

Structural and diffusion-weighted images were acquired in 139 preschool-aged children with ASD (112 males/27 females) and 82 typically developing (TD) controls (53 males/29 females). Longitudinal scanning at two additional annual time points was carried out in a subset of these participants. Callosal organization was evaluated using two approaches: 1) diffusion tensor imaging (DTI) tractography to define subregions based on cortical projection zones and 2) as a comparison to previous studies, midsagittal area analysis using Witelson subdivisions. Diffusion measures of callosal fibers were also evaluated.

**Results:**

Analyses of cortical projection zone subregions revealed sex differences in the patterns of altered callosal organization. Relative to their sex-specific TD counterparts, both males and females with ASD had smaller regions dedicated to fibers projecting to superior frontal cortex, but patterns differed in callosal subregions projecting to other parts of frontal cortex. While males with ASD had a smaller callosal region dedicated to the orbitofrontal cortex, females with ASD had a smaller callosal region dedicated to the anterior frontal cortex. There were also sex differences in diffusion properties of callosal fibers. While no alterations were observed in males with ASD relative to TD males, mean diffusivity (MD), axial diffusivity (AD), and radial diffusivity (RD) were all increased in females with ASD relative to TD females. Analyses of Witelson subdivisions revealed a decrease in midsagittal area of the corpus callosum in both males and females with ASD but no regional differences in specific subdivisions. Longitudinal analyses revealed no diagnostic or sex differences in the growth rate or change in diffusion measures of the corpus callosum from 3 to 5 years of age.

**Conclusions:**

There are sex differences in the pattern of altered corpus callosum neuroanatomy in preschool-aged children with ASD.

**Electronic supplementary material:**

The online version of this article (doi:10.1186/s13229-015-0005-4) contains supplementary material, which is available to authorized users.

## Background

The neuropathology of autism spectrum disorder (ASD) involves the abnormal development of white matter and brain connectivity [1,2]. The corpus callosum is the largest fiber bundle in the brain and consists of well-organized neocortical commissural connections [3]. In older children, adolescents and adults with ASD, the corpus callosum is consistently reported to be smaller, with decreased fractional anisotropy [4] and reduced interhemispheric functional connectivity [5]. Fewer studies, however, have evaluated callosal deficits in young children with ASD [6-8], and very little is known about sex differences in the corpus callosum in ASD [9]. We sought to evaluate the corpus callosum in preschool-aged children with ASD and to determine whether there are differences in callosal organization between males and females with ASD.

ASD is much more common in males than females [10,11], and females remain underrepresented in research studies. Consequently, little is known about the neuropathology of ASD in females and whether sex differences exist. Emerging evidence suggests that females with ASD have a different neuroanatomical profile than males [12-15], including in the corpus callosum [9,13]. We sought to extend these findings in our relatively large cohort of preschool-aged children. We conducted a longitudinal study of the development of the corpus callosum in children with ASD from 3 to 5 years of age using structural and diffusion-weighted imaging. The cohort includes 139 children with ASD (112 males/27 females) and 82 typically developing (TD) control children (53 males/29 females) enrolled in the Autism Phenome Project.

Callosal axons carry information between many different parts of the neocortex. Bundles of callosal axons that project to different regions of the cortex are fairly large and can be identified and measured using modern diffusion-weighted imaging and tractography in individual subjects [16,17]. Using these methods, we segmented the corpus callosum based on cortical projection zones and examined the diffusion properties of the axon bundles that innervate specific cortical regions [17]. As a comparison to previous studies, we also evaluated the midsagittal area of the corpus callosum using standard Witelson subdivisions [18].

## Methods

### Participants

Participants were enrolled in the University of California (UC) Davis MIND Institute Autism Phenome Project. This study was approved by the UC Davis Institutional Review Board. Informed consent was obtained from the parent or guardian of each participant. Structural and diffusion-weighted images (*n* = 397) were acquired for at least one time point in 221 children (139 ASD, 82 TD). Of these, 98 children (60 ASD [47 males/13 females], 38 TD [25 males/13 females]) were imaged at one time point and 123 children had longitudinal magnetic resonance imaging (MRI) data available: 70 (46 ASD [35 males/11 females], 24 TD [15 males/9 females]) were imaged at two time points, and 53 (33 ASD [30 males/3 females], 20 TD [13 males/7 females]) were imaged at all three time points. Data from a subset of these participants have been reported previously [14,19].

Diagnostic assessments included the Autism Diagnostic Observation Schedule-Generic (ADOS-G) [20,21] and the Autism Diagnostic Interview-Revised (ADI-R) [22]. All diagnostic assessments were conducted or directly observed by trained, licensed clinical psychologists who specialize in autism and had been trained according to research standards for these tools. Inclusion criteria for ASD were taken from the diagnostic definition of ASD in young children formulated and agreed upon by the Collaborative Programs of Excellence in Autism (CPEA) using DSM-IV criteria. Participants met ADOS cutoff scores for either autism or ASD. In addition, they exceeded the ADI-R cutoff score for autism on either the Social or Communication subscale and within two points of this criterion on the other subscale. An ADOS severity score was calculated ranging from 1 to 10 [23], which allows comparison of autism severity across participants tested with different ADOS-G modules. Overall developmental quotients (DQ) were determined for all participants using the Mullen Scales of Early Development (MSEL) [24].

Typically developing children were screened and excluded for ASD using the Social Communication Questionnaire [25]. Children with typical development were also excluded if they had first-degree relatives (that is, siblings) with ASD. Inclusion criteria included developmental scores within two standard deviations on all scales of the MSEL. All children, both TD controls and children with ASD, were native English speakers, ambulatory, had no contraindications for MRI, no suspected vision or hearing problems or known genetic disorders, or other neurological conditions. In the ASD group, one child was excluded for the presence of fragile X.

### Imaging

MRI scans were acquired during natural, nocturnal sleep [26] at the UC Davis Imaging Research Center on a 3T Siemens Trio whole-body MRI system (Siemens Medical Solutions, Erlangen, Germany) using an 8-channel head coil (Invivo Corporation, Gainesville, FL, USA). Images were obtained using a three-dimensional T1-weighted magnetization-prepared rapid acquisition gradient-echo (MPRAGE) sequence (TR 2,170 ms; TE 4.86 ms; matrix 256 × 256; 192 slices in the sagittal direction; 1.0-mm isotropic voxels) and a diffusion-weighted, spin echo, echo planar imaging sequence (‘ep2d_diff’; number of slices: 72; slice thickness: 1.9 mm; slice gap: 0.0; matrix size: 128 × 128; voxel size: 1.9-mm isotropic; phase-encoding direction: anterior to posterior (A>>P); TR: 11,500; TE: 91; scan time: 6 min and 29 s), with an effective *b*-value of 700 mm^2^/s, 30 gradient directions, and five interleaved *b* = 0 images. Thirty-six children (24 ASD [22 males/2 females], 12 TD [10 males/2 females]) were excluded from the study due to waking up prior to the completion of the diffusion sequence.

To accomplish longitudinal imaging at three time points, scans were acquired from October 2007 to October 2012. In August 2009, the Siemens 3T Trio MRI system was upgraded to a Trio Total Imaging Matrix (TIM) MRI System running version VB15A operating system software. All the VA25A sequences were upgraded and mapped to their corresponding VB15A sequences.

For T1-weighted scans, changes in hardware and software over this scanning period were controlled for using a calibration phantom (ADNI MAGPHAM, The Phantom Laboratory, Salem, NY, USA, http://www.phantomlab.com) scanned at the end of each MRI session. Distortion correction was then carried out on each participant’s MPRAGE image (Image Owl, Inc., Greenwich, NY, USA, http://www.imageowl.com/) [19]. This step ensures accuracy in measurements of the midsagittal area of the corpus callosum and total cerebral volume by removing any distortion associated with changes in scanner hardware over time.

For the diffusion-weighted sequence, the spatial resolution, *b*-value, and gradient directions were preserved following the MRI system upgrade. While the diffusion-weighted parameters were not directly changed, there may be differences in diffusion-weighted measures in regions with reduced geometric distortion. To control for these differences, we include MRI system upgrade status (pre-upgrade vs. post-upgrade) as a nuisance covariate for all statistical analyses involving diffusion tractography or diffusion-weighted measures.

In addition, we evaluated the proportion of participants (by diagnosis and sex) scanned pre- vs. post-upgrade. Prior to the upgrade, 126 (74 ASD [59 males/15 females], 52 TD [37 males/15 females]) scans were acquired. After the upgrade, 271 (177 ASD [148 males/29 females], 94 TD [57 males/37 females]) scans were acquired. Importantly, there were no differences across scanner upgrade status for diagnostic group (chi-square = 1.6, *P* = 0.21) or sex (chi-square = 0.01, *P* = 0.91). Within each diagnostic group, there was also no difference in observed frequencies between males and females (ASD: chi-square = 0.55, *P* = 0.46, TD: chi-square = 1.6, *P* = 0.20).

For participants who were scanned at multiple time points, we also evaluated diagnoses and sex of participants whose longitudinal scanning took place entirely pre-scanner upgrade, those that spanned the upgrade point, and those whose scanning was entirely post-upgrade. Of the 123 participants with longitudinal data, 9% (7 ASD [5 males/2 females], 4 TD [3 males/1 female]) have complete pre-scanner upgrade data, 43% (31 ASD [26 males/5 females], 22 TD [13 males/9 females]) span the upgrade point, and 48% (41 ASD [34 males/7 females], 18 TD [12 males/6 females]) have complete post-scanner upgrade data. There were no differences in the proportion of participants scanned either pre-, post-, or spanning upgrade status for diagnostic group (chi-square = 1.47, *P* = 0.48) or sex (chi-square = 0.35, *P* = 0.84). Within each diagnostic group, there were also no differences across sexes (ASD: chi-square = 0.63, *P* = 0.73, TD: chi-square = 0.491, *P* = 0.78).

### DTI image processing

Raw diffusion images were checked for the presence of motion artifacts prior to preprocessing. Each image was visually inspected, and volumes were excluded if any signal dropout was detected. The number of volumes excluded was recorded, and if the number of diffusion directions excluded was greater than or equal to six (20% of total diffusion directions), the entire scan was excluded. By these criteria, 14 scans (4 ASD [4 males/0 female], 10 TD [3 males/7 females] were excluded for too much motion. The remaining 397 scans were included in the analysis. Of these, 289 (73%) contained no artifacts - all diffusion directions were included. In 47 scans (12%), one diffusion direction was excluded. This most frequently occurred at the beginning of the sequence - some children would startle in their sleep at the onset of the noises. Two to three diffusion directions (volumes) were excluded in an additional 50 scans (12.6%), and four to six diffusion directions (volumes) were excluded in 11 scans (3%). Additional file 1: Table S1 provides details about the number of volumes excluded for each diagnostic group and across sexes. Importantly, the number of excluded volumes (0 to 6) did not differ by diagnostic group (Fisher’s exact test, *P* = 0.13) or sex (Fisher’s exact test, *P* = 0.16). Within each diagnostic group, ASD or typical development, Fisher’s exact test revealed no differences by sex (ASD: *P* = 0.16, TD: *P* = 0.44).

Diffusion tensor imaging (DTI) data were preprocessed and analyzed using mrDiffusion, a custom, freely available software package developed by the Vision, Imaging Science and Technology Activities (VISTA) lab, Stanford, CA, USA (http://vistalab.stanford.edu/newlm/index.php/Software). DTI preprocessing included removal of eddy current distortion effects [27], alignment to the T1 image in AC/PC space, and calculation of diffusion tensors. Artifacts were removed using the robust estimation of tensors by outlier rejection (RESTORE) algorithm [28].

### Tractography of callosal fibers and segmentation by cortical projection zone

**Figure 1:**
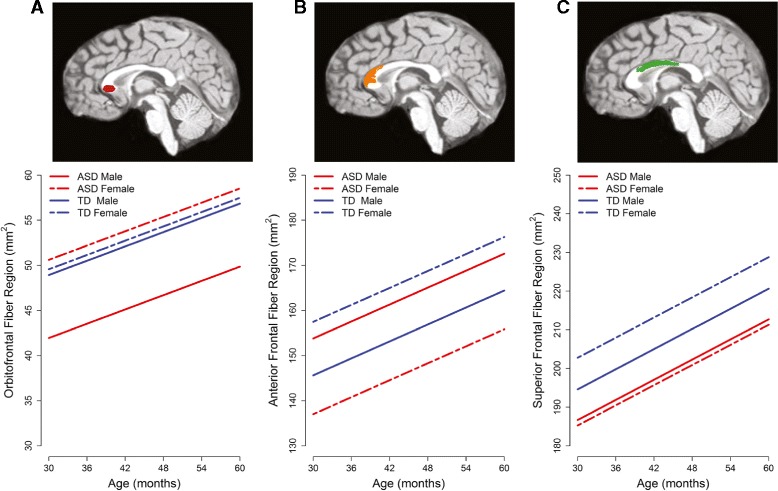
**Sex differences in cortical projection zone subregions across three MRI time points.** Estimated trajectories depicted were estimated for TCV equal to the time 1 average for TD children before the scanner upgrade. **(A)** The orbitofrontal fiber region is smaller in males with ASD than in TD males but does not differ in females. **(B)** The anterior frontal fiber region is smaller in females with ASD than in TD females. In males, the opposite pattern is observed; males with ASD are larger than TD males. **(C)** The superior frontal fiber region is smaller in both males and females with ASD than TD counterparts, though the difference is larger in females.

For fiber tractography, an ROI was defined manually in mrDiffusion by tracing the corpus callosum on a single slice in the midsagittal plane. Fiber tracts in the left and right hemisphere were then estimated separately using a deterministic streamlined tracking algorithm [29-31] with a fourth-order Runge-Kutta path integration method. Step size was fixed at 1 mm and path tracing proceeded using a fractional anisotropy (FA) threshold of 0.15 and a path angle threshold of 30°. The subset of fibers in each hemisphere intersecting the corpus callosum ROI was identified (Figure 1A). Using these sets of fibers, the callosum was segmented for each hemisphere separately according to the fiber projection zone using the method introduced by Huang *et al*. [17]. In brief, fibers were visualized using Quench (http://white.stanford.edu/newlm/index.php/QUENCH), and a series of planes were used to define anatomical targets of the callosal fibers [16]. A total of 397 scans from 221 participants were analyzed. Five trained raters manually segmented the callosal fibers. Intraclass correlation coefficients (ICCs) were calculated for each fiber region and ranged from 0.80 to 0.99. Mean ICCs for the left and right hemispheres were 0.96 and 0.93. In addition, a single expert rater (CWN) reviewed and edited segmentations for all 794 hemispheres. Defined projection zones included orbitofrontal, anterior frontal, lateral frontal, superior frontal, superior parietal, posterior parietal, occipital, and temporal regions (see Figure 1A,B,C). The cross-sectional area of each cortical projection zone fiber subdivision was determined on the midsagittal plane (Figure 1D). To evaluate diffusion properties, fibers from the right and left hemispheres were merged and cropped to the high coherence zone of 1 cm within the midsagittal plane (Figure 1E). Mean diffusivity (MD), radial diffusivity (RD), axial diffusivity (AD), and FA were measured for each fiber subdivision. In brief, AD describes diffusion parallel to the principle diffusion direction (that is, along the long axis of an axonal bundle), and RD describes diffusion perpendicular to the principle diffusion direction. MD describes the average total diffusion, and FA is a scalar value of the normalized standard deviation of the three diffusion directions.

**Figure 2:**
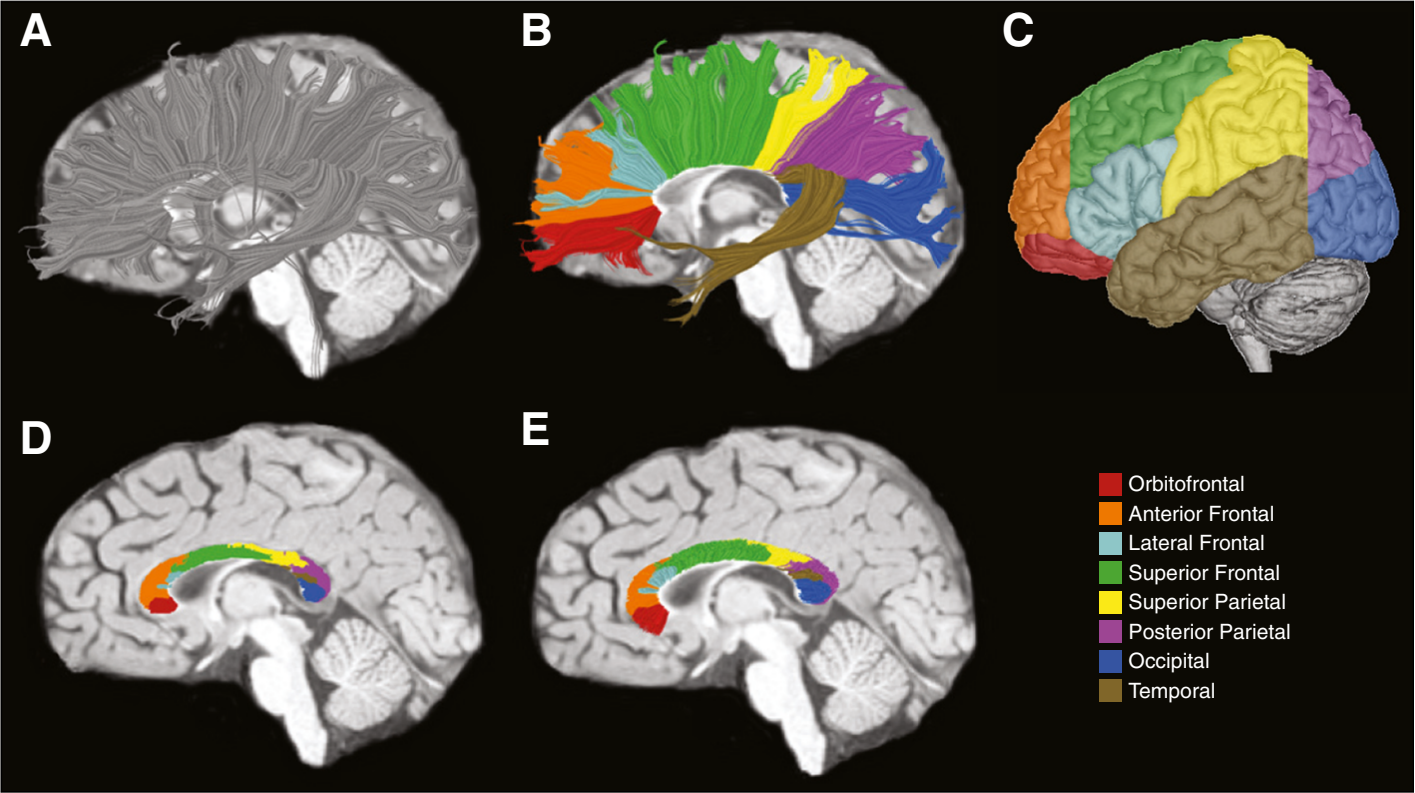
**Segmenting the corpus callosum based on cortical projection zones using DTI tractography. (A)** Callosal fibers are tracked separately for each hemisphere. **(B)** Callosal fibers are then segmented based on fiber termination points in eight anatomically defined cortical projection zones (see legend at lower right). **(C)** Cortical projection zones displayed on the lateral surface of the left hemisphere. **(D)** Cross-sectional areas for each subregion are determined on the midsagittal slice. **(E)** Callosal fibers for each hemisphere are merged and clipped at 1 cm, a zone of high coherence. Diffusion properties are determined for these fiber segments.

### Corpus callosum midsagittal area and Witelson subdivisions

Distortion-corrected T1-weighted images were preprocessed to remove non-brain tissue and to correct for field inhomogeneity [32]. Total cerebral volume (TCV) was derived as described previously [14,19]. For midsagittal corpus callosum measurements, images were aligned along the axis of the anterior and posterior commissures (AC/PC) and resampled to yield 0.5-mm^3^ voxels using Analyze 11.0 [33]. The midsagittal slice was defined using the central fissure and the aqueduct of sylvius. The midsagittal area of the corpus callosum was manually delineated by two expert raters. ICCs for subdivisions ranged from 0.83 to 0.97. ICC for the total corpus callosum was 0.98. After the total midsagittal area of the corpus callosum was defined, seven subdivisions were segmented according to the procedure described by Witelson (1989) [18]. Subdivisions included rostrum, genu, rostral body, anterior midbody, posterior midbody, isthmus, and splenium [18].

### Analytic plan

We used mixed-effect regression models for repeated measures [34] to characterize the longitudinal changes in the corpus callosum and to examine the association of sex, diagnosis, and different callosal subregions/subdivisions with overall levels and rates of change in callosal size, while accounting for the effect of other variables such as TCV or scanner upgrade. The models are flexible and allow children to have different numbers of scans and different lag times between the scans. This approach allowed us to treat subregion/subdivision as a repeated effect within the mixed-effect models for the corpus callosum. The core model used for the cortical projection zone subregions had fixed effects for subregion (orbital, anterior frontal, lateral frontal, superior frontal, superior parietal, posterior parietal, temporal, occipital), diagnosis, sex, upgrade status (pre- or post-upgrade), age, and TCV. Both age and TCV were centered at the time 1 averages for the TD control subjects. In this way, the intercept in the model can be interpreted as the average occipital subregion area (the reference region) for a TD female with average age and TCV at time 1. Individuals were permitted to have differing overall and subregion sizes, by including random effects for intercept and subregions that were assumed to follow a multivariate normal distribution. We allowed the variance of the residuals to differ across cortical projection zone subregions.

This core model allowed us to describe the overall pattern of differences across regions, diagnosis, and sex and assess maturation (age) effects. We then built a hierarchy of questions by adding and testing all two-way interactions between age, subregion/subdivision, sex, and diagnosis in the model. This allowed us to assess whether the maturation effects differed by subregion, sex, or diagnosis, whether the pattern of regional differences differed by sex or diagnosis, and whether there was a sex by diagnosis interaction. These interactions were not retained in the final model when they failed to add significantly to the model. Higher-order three-way or four-way interaction effects were likewise tested against simpler models including all relevant significant lower-order interaction effects. Any significant interaction effects including diagnosis by sex were further examined for a subset of specific simple comparisons of interest involving simple comparisons of diagnosis within levels of sex (males: ASD vs. TD, females: ASD vs. TD) and simple comparisons of sex within levels of diagnosis (ASD: males vs. females, TD: males vs. females). As such, alpha levels were not adjusted for such simple effect testing given the limited number of comparisons that were considered of interest *a priori*.

Similar mixed-effect models were used to model diffusion-weighted measures (FA, MD, RD, and AD) and Witelson subdivisions. The models for diffusion measures were adjusted for scanner upgrade status (pre- or post-upgrade) but not for TCV. The model for Witelson subdivisions included TCV.

Secondary analyses examined whether the results of the primary analyses could be accounted for by baseline DQ. All models were implemented using PROC MIXED in SAS 9.4 [35].

## Results

### Participant characteristics

Structural and diffusion-weighted images (*n* = 397) were collected in 139 children with ASD (112 males/27 females) and 82 TD children (53 males/29 females) for at least one of the three annual MRI time points centered at ages 36.3 months (range: 25.7 to 47.5), 50.0 months (range: 37.4 to 65.5), and 64.0 (range: 52.8 to 81.6) months. Table 1 provides participant characteristics at each of the MRI time points. Groups were well matched in age across all three MRI time points. As expected, TD children had higher DQ than ASD children. There were no differences in DQ or ADOS severity across males and females with ASD.

**Table 1:** **Participant characteristics across the three scanning time points**

	**Time 1**				**Time 2**				**Time 3**			
	**ASD**		**TD**		**ASD**		**TD**		**ASD**		**TD**	
	**Male**	**Female**	**Male**	**Female**	**Male**	**Female**	**Male**	**Female**	**Male**	**Female**	**Male**	**Female**
*n*	97	21	44	25	76	15	30	15	34	8	20	12
Age	36.5 (5.6)	37.4 (5.0)	35.98 (5.1)	34.9 (4.5)	49.8 (5.7)	51.0 (5.5)	49.1 (5.0)	51.3 (6.1)	63.5 (5.5)	63.5 (4.0)	64.7 (7.7)	64.9 (1.8)
DQ	64.7 (22.1)	66.7 (21.5)	106.4 (13.0)	108.6 (10.8)	--		--		--		--	
ADOS severity score	7.97 (1.7)	7.71 (1.8)	--		--		--		--		--	

Data are expressed as mean (standard deviation). DQ, developmental quotient; ADOS, Autism Diagnostic Observation.

### Cortical projection zone subregions

Additional file 2: Table S2 presents detailed results from the final mixed-effect model for cortical projection zone subregions. In summary, there was a significant main effect for TCV (*P* < 0.001) but not for scanner upgrade status (*P* = 0.32). The interaction between age and subregions was significant (*P* < 0.0001), indicating that subregions grew at different rates, but there were no significant age by diagnosis or age by sex interaction effects (both *P* > 0.19), indicating that the growth rate did not differ between diagnosis and sex. There was a significant three-way interaction effect between diagnosis, cortical projection zone subregion, and sex (*P* = 0.004). Simple effects for subregion-specific diagnosis and sex differences are detailed in Table 2. Subregions that differ by diagnosis and sex include the orbitofrontal, anterior frontal, and superior frontal regions. Differences are depicted in Figure 2. Specifically, the orbitofrontal fiber region area is decreased in males with ASD relative to TD males (estimated difference = −6.98, *P* = 0.02) but did not differ between females with ASD and TD females (*P* = 0.83). In contrast, the anterior frontal and superior frontal fiber regions are significantly decreased in females with ASD compared to TD females (anterior frontal: estimated difference = −20.45, *P* = 0.01; superior frontal: estimated difference = −17.49, *P* = 0.01). In males, there were marginally significant differences in these regions with differing patterns. In the anterior frontal region, males with ASD were increased relative to TD males (estimated difference = 8.18, *P* = 0.09), opposite to the pattern observed in females. In the superior frontal region, the pattern was similar to females; males with ASD were decreased relative to TD males (estimated difference = −7.92, *P* = 0.07). There was also a marginally significant difference in the posterior parietal fiber region area, with females with ASD decreased relative to TD females (estimated difference = −13.34, *P* = 0.07) but no difference between males with ASD and TD males (*P* = 0.22).

**Table 2:** **Estimated diagnosis and sex differences across cortical projection zone subregions**

	**Diagnosis differences by sex**				**Sex difference by diagnosis**			
	**Males: ASD vs. TD**		**Females: ASD vs. TD**		**ASD: males vs. females**		**TD: males vs. females**	
	**Estimate (SE)**	***P*** **value**	**Estimate (SE)**	***P*** **value**	**Estimate (SE)**	***P*** **value**	**Estimate (SE)**	***P*** **value**
Subdivision								
Orbitofrontal	−6.98 (2.93)	0.02	1.04 (4.67)	0.82	−8.66 (3.84)	0.02	−0.64 (4.05)	0.88
Anterior frontal	8.17 (4.81)	0.09	−20.45 (7.81)	0.01	16.77 (6.37)	0.01	−11.85 (6.67)	0.08
Lateral frontal	−3.18 (5.28)	0.55	8.24 (7.97)	0.30	−11.23 (6.58)	0.09	0.18 (6.70)	0.98
Superior frontal	−7.92 (4.36)	0.07	−17.49 (7.02)	0.01	1.40 (5.73)	0.81	−8.17 (6.02)	0.18
Superior parietal	−2.92 (3.81)	0.44	−6.91 (6.09)	0.26	−5.11 (4.99)	0.31	−9.10 (5.25)	0.08
Posterior parietal	5.47 (4.49)	0.22	−13.34 (7.23)	0.07	15.75 (5.90)	0.01	−3.06 (6.20)	0.62
Temporal	−4.06 (3.70)	0.27	1.89 (6.02)	0.75	−5.69 (4.92)	0.26	0.27 (5.15)	0.96
Occipital	−2.30 (3.01)	0.44	−5.73 (4.80)	0.23	−2.66 (3.95)	0.50	−6.09 (4.16)	0.14

Parameter estimates and standard errors are from the mixed-effects models assessing the relationship of diagnostic group, age, cortical projection zone subregions, and sex with diffusion measures. The reported models included fixed effects for diagnosis, sex, age, scanner upgrade, TCV, subregion, the three-way interaction between subregion, sex, and diagnosis, as well as all two-way interactions involving subregion, sex, and diagnosis, and the two-way interaction between age and subregion. Random effects for person and region were included to account for the repeated measures. Models also included and tested the rest of the two-way interactions involving age, sex, and diagnosis, but none were significant and were not retained in the reported model.

There were between sex differences for males and females with ASD in orbitofrontal, anterior frontal, and posterior parietal fiber region areas (all *P* < 0.05). There were only marginally significant differences between TD males and females in the anterior frontal and superior parietal regions (*P* = 0.08).

A mixed-effect regression model fitted in secondary analyses to test for the effects of DQ revealed no effect for baseline DQ (*P* = 0.53).

### Diffusion-weighted measures

**Figure 3:**
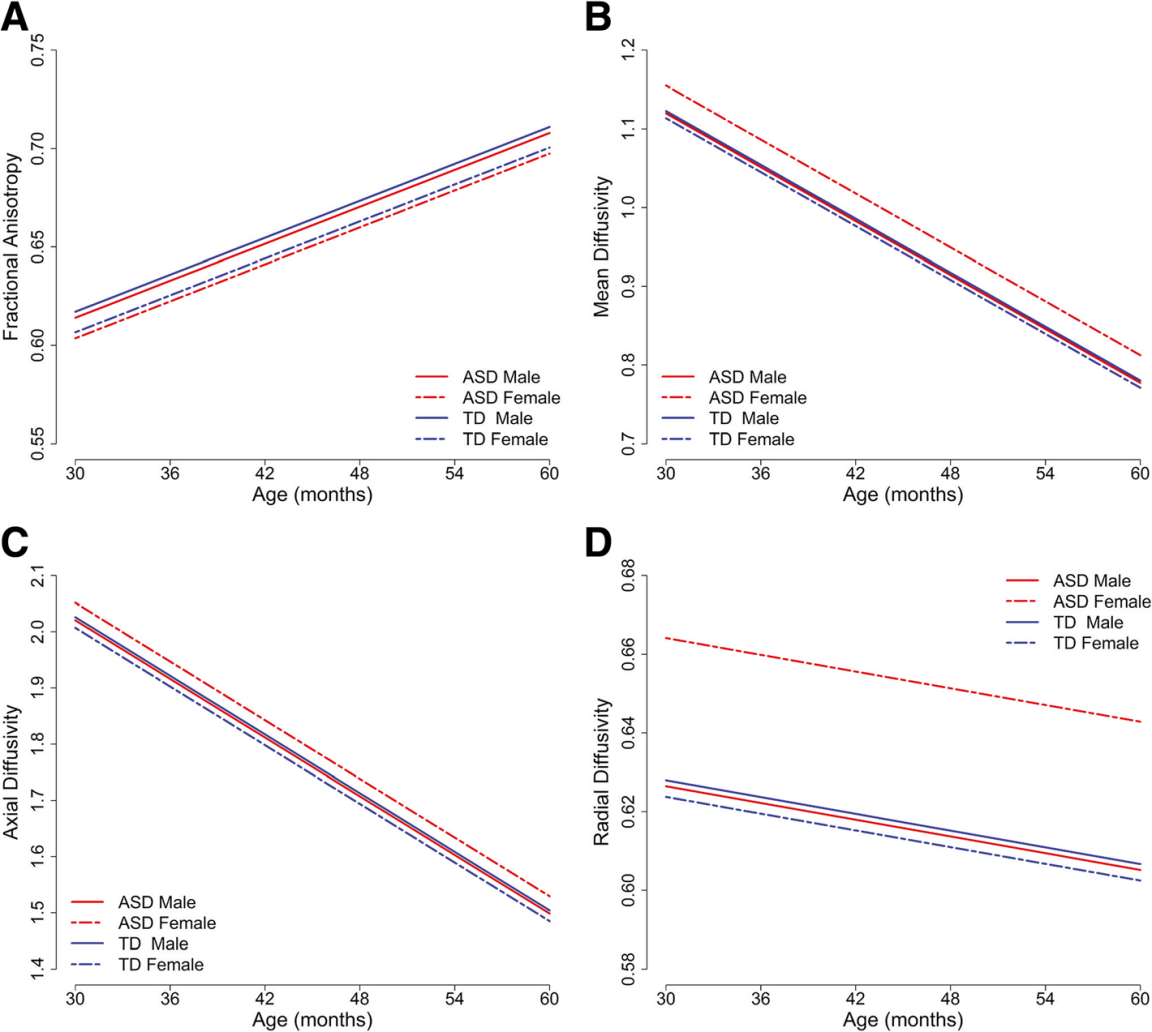
**Diffusion measures of callosal fibers across three MRI time points.** The reference cortical projection zone subregion (occipital) is depicted, which is representative of all subregions given that there were no diagnosis or sex interactions with subregion. **(A)** FA is higher in males than in females both ASD and TD groups. There were no differences in FA across ASD and TD. **(B-D)** MD, AD, and RD were all increased in females with ASD relative to TD females. Males with ASD did not differ from TD males.

Additional file 3: Table S3 presents detailed results from the final mixed-effect models for diffusion-weighted measures. For all diffusion measures, there was a significant main effect for scanner upgrade status (all *P* < 0.0001) and a significant age by region interaction effect (all *P* < 0.0001), but there were no significant interactions between diagnosis and age or diagnosis and subregion, indicating that diagnosis differences in FA, MD, RD, and AD did not vary with age or cortical projection zone subregion. For FA, there was a significant main effect for sex (*P* = 0.03), but not diagnosis (*P* = 0.47). Males had higher FA than females in both ASD and TD children (Figure 3A). For MD, RD, and AD, there was a significant diagnosis by sex interaction (all *P* < 0.03). Table 3 presents estimated diagnosis and sex differences from the final mixed-effect models for these three measures. As seen in Figure 3B,C,D, MD, RD, and AD are increased (*P* < 0.01) in females with ASD relative to TD females, but not in males with ASD relative to TD males. In addition, the same pattern of increased MD, RD, and AD was significant in females with ASD relative to males with ASD (*P* < 0.02). The other simple effect comparisons for TD males vs. TD females were not significant for MD, RD, and AD.

**Table 3:** **Estimated diagnosis and sex differences for diffusion measures**

	**Diagnosis differences by sex**				**Sex difference by diagnosis**			
	**Males: ASD vs. TD**		**Females: ASD vs. TD**		**ASD: males vs. females**		**TD: males vs. females**	
	**Estimate (SE)**	***P*** **value**	**Estimate (SE)**	***P*** **value**	**Estimate (SE)**	***P*** **value**	**Estimate (SE)**	***P*** **value**
Diffusion parameter								
Mean diffusivity (10^−5^ mm^2^/s)	−0.30 (0.88)	0.74	4.14 (1.41)	0.003	−3.51 (1.13)	0.002	0.92 (1.22)	0.45
Axial diffusivity (10^−5^ mm^2^/s)	−0.55 (1.01)	0.59	4.44 (1.62)	0.006	−3.09 (1.30)	0.02	1.90 (1.40)	0.17
Radial diffusivity (10^−5^ mm^2^/s)	−0.15 (0.99)	0.88	4.04 (1.58)	0.01	−3.76 (1.27)	0.003	0.42 (1.37)	0.76

Parameter estimates and standard errors are from the mixed-effects models assessing the relationship of diagnostic group, age, cortical projection zone subregions, and sex with diffusion measures. The reported models included fixed effects for scanner upgrade, diagnosis, sex, age, subregion, and the interactions between diagnosis and sex and between age and subregion. Random effects for person and region were included to account for the repeated measures. Models also included and tested other two-way interactions, but none were significant and were not retained in the reported model.

Similar to the results for cortical projection zone subregions, adding baseline DQ to the models did not change the results and DQ did not emerge as a significant predictor for any of the diffusion-weighted measures (all *P* > 0.71).

### Witelson subdivisions

As detailed in Additional file 4: Table S4, there were significant main effects for TCV (*P* < 0.001), age (*P* < 0.001), and diagnosis (*P* = 0.047). The midsagittal area of the corpus callosum was smaller in all children with ASD relative to their TD counterparts. However, there was no diagnosis by subdivision interaction (*P* = 0.90), indicating that the pattern of differences between different subdivisions was similar across diagnoses. There was no diagnosis by age interaction (*P* = 0.83), indicating that growth rates did not differ between ASD and TD, and there was no diagnosis by sex interaction (*P* = 0.29). Secondary analyses testing the effect of DQ revealed no effect for baseline DQ (*P* = 0.97).

## Discussion

The aims of this study were to evaluate the corpus callosum in ASD and to assess whether there are sex differences in callosal organization in ASD. We examined callosal size and organization of fibers projecting to cortical targets. We also evaluated diffusion characteristics of callosal fibers. Overall, the results suggest sex differences in the pattern of alterations in the corpus callosum of preschool-aged children with ASD. Specifically, the organization of callosal fibers projecting to the frontal lobe was different in males and females with ASD relative to their typically developing counterparts. While both males and females with ASD had smaller regions of the corpus callosum with fibers directed to superior frontal cortex, only males with ASD had a smaller region with fibers directed to the orbitofrontal cortex. In contrast, females with ASD had a smaller region of the corpus callosum associated with the anterior frontal cortex.

We also observed that diffusion measures were more altered in females with ASD than males with ASD. While females with ASD exhibited increases in AD, RD, and MD relative to TD females, males with ASD did not differ from TD males on any diffusion measures. The alterations in diffusion measures observed in females with ASD could reflect changes in axonal membrane integrity, delayed or decreased myelination, or increased intracellular space with fewer and/or thinner axons [36-40].

As a comparison to previous studies, we also conducted analyses of the midsagittal area of the corpus callosum using Witelson subdivisions. Although we did not observe any differences in the size of individual subdivisions between either males or females with ASD and their TD counterparts, we did observe an overall reduction in midsagittal area in both males and females with ASD, which is consistent with one other study in 3- to 4-year-old children [4].

The longitudinal nature of this study allowed us to evaluate the development of the corpus callosum between 3 and 5 years of age. However, we did not detect any differences in the rate of growth of the corpus callosum or change in diffusion measures between ASD and TD children. This suggests that the observed differences in corpus callosum size, fiber organization, and microstructure in ASD were established prior to 3 years of age. A recent prospective study of the development of white matter tracts in infants who later develop autism suggests that aberrations in the trajectory of white matter development in ASD may occur as early as the first year of life [41].

**Figure 4:**
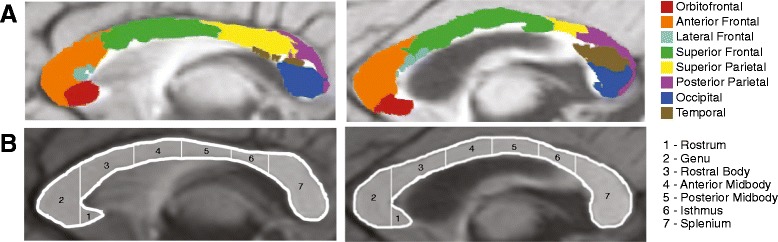
**Comparison of (A) cortical projection zone subregions and (B) Witelson subdivisions in an ASD (left) and TD (right) example.** Although there is some overlap between the two parcellation methods, there is also a high degree of variability, particularly in the cortical projection zone segmentation. Using cortical projection zones provides a finer-grained anatomic parcellation of the corpus callosum.

To our knowledge, this is the first study of young children with ASD to evaluate subregions of the corpus callosum based on anatomically defined cortical projection zones. Subdividing the corpus callosum using the Witelson protocol has yielded somewhat inconsistent results, which may be due, in part, to the limited anatomical specificity in Witelson subdivisions and individual variability in callosal organization. Figure 4 depicts examples of the two methods of evaluating callosal organization. There is considerable variability in the cortical projection zone pattern that is not captured by Witelson subdivisions. Arguably, analysis based on the pattern of projections to distinct cortical regions is more sensitive to disorders that are highly associated with frontal lobe function. It should be noted, however, that diffusion tractography has some inherent limitations, including the inability to resolve white matter tracts that are crossing (for example, lateral projections of the callosum that intersect with corona radiata).

Nevertheless, there is overlap between our results and previous studies that have utilized Witelson subdivisions. In their meta-analysis of older individuals with ASD, Frazier and Hardan report that the most impacted portion of the corpus callosum in ASD is the rostral body and that differences generally decrease in the more caudal subdivisions [4], and in a study of 3 to 4 years old, the greatest changes were observed in the rostrum and rostral body [7]. The results from the present study generally support these findings. Although we did not observe differences based on analyses of Witelson subdivisions, we did observe differences in both males and females with ASD in callosal regions that contain fibers that project to frontal lobe regions, which correspond to the more rostral Witelson subdivisions.

In general, DTI studies of the corpus callosum in older individuals with ASD report decreased FA and increased MD, which appears to be driven by increases in radial rather than axial diffusivity [42]. However, in two studies of 1.8- to 3.3-year-old and 1.5- to 5.8-year-old children with ASD, Ben Bashat and colleagues found increased FA in the corpus callosum [6,8]. This has been interpreted as an indication of precocious development of white matter associated with the neocortex. In our study of 3 to 5 years old, we did not observe any differences in FA in either males or females with ASD, but we did observe increased MD, RD, and AD in females, but not males with ASD. Several factors may contribute to these seemingly discrepant results. Importantly, the age range in the previous studies is younger than in the current sample, including toddlers as young as 1.5 years old. It is also not clear whether the previous studies included mixed-sex samples. The recent prospective study by Wolff and colleagues suggests that FA is higher in children with ASD around 6 to 12 months of age but then is lower in toddlers at 2 years of age [41]. Thus, the aforementioned DTI studies reporting increases in FA in young children with ASD may be capturing the earlier part of that developmental trajectory, while our study emphasizes the latter part. Additional prospective longitudinal studies that span infancy to early childhood are needed.

In typical development, the size of the corpus callosum does not appear to be sexually dimorphic [43-46], and our current results are consistent with this finding; there were no differences in callosal size between typically developing males and females after adjusting for total brain volume. In contrast, we did observe sex differences in the pattern of callosal alterations between males and females with ASD relative to their typically developing counterparts. Autism is much more common in boys than in girls, and although this disparate sex ratio is among the most highly replicated findings in studies of ASD [10,11], sex differences in ASD neuropathology remain poorly understood. Historically, females with ASD have been underrepresented in research studies due to the strong male bias of ASD.

It has long been theorized that a multifactorial liability model may explain the sex bias in ASD [47-49]. This model suggests that there are multiple genetic and environmental factors (that is, etiologic load) that contribute to an individual’s liability for ASD and that the threshold is shifted in females such that a higher etiologic load is needed for females to meet criteria for ASD (the so-called ‘female protective effect’). There is some preliminary evidence that preschool-aged females with ASD show a different and perhaps more extreme pattern of neural abnormalities in the amygdala, temporal lobe, and cerebellum [12,50]. But many previous studies of the corpus callosum have either included only males [51,52] or very small samples of females [7,53]. There is some evidence for sex differences in the corpus callosum in adults with ASD [9,13], but additional studies are needed. Our sample size of 27 females with ASD is among the larger sample sizes in the MRI literature of ASD, and our results provide a striking example of sex differences in the neuropathology of ASD.

However, the functional consequence of the different patterns of callosal organization in males and females with ASD remains unclear. The orbitofrontal cortex is involved in emotional processing and decision-making for reward-related processes [54]. The anterior frontal cortex, comprised of the frontal pole and rostral portions of the superior and middle frontal gyri, is involved in higher-order executive function and cognitive processes [55]. Both regions have been implicated in the neuropathology of autism [56-58], but sex-specific differences in the organization of the frontal lobe have not yet been explored. One possibility is that alterations in orbitofrontal interhemispheric connectivity in boys with ASD may lead to greater affective disturbances and a lower liability threshold for ASD (that is, easier detectability). In contrast, altered anterior frontal interhemispheric connectivity in females with ASD may manifest as higher-level cognitive disturbances that contribute to the ‘female protective effect’ and a higher liability threshold, making ASD more difficult to detect. Additional studies are under way to relate these findings to the behavioral manifestations of ASD. Future studies are also needed to determine whether these sex differences in the pattern of callosal organization persist as the child matures.

## Conclusions

We have identified sex differences in the pattern of alterations in the fiber organization and microstructural characteristics of the corpus callosum in 3- to 5-year-old children with ASD. These results indicate that males and females with ASD should be evaluated separately. Further investigations using sex-balanced ASD cohorts are necessary to fully explore sex differences in the neural phenotypes of ASD.

## Additional files

Additional file 1: Table S1. Assessment of head motion in diffusion-weighted images. Raw counts and percent of the participants with excluded volumes. Additional file 2: Table S2. Summary (parameter estimates and standard errors) of the random-effect models assessing the relationship of diagnostic group, sex, age, and cortical projection zone subregions with midsagittal area. Additional file 3: Table S3. Summary (parameter estimates and standard errors) of the random-effect models assessing the relationship of diagnostic group, sex, and age with diffusion parameters. Additional file 4: Table S4. Summary (parameter estimates and standard errors) of the random-effect model assessing the relationship of diagnostic group, sex, age, and Witelson subdivision with corpus callosum area (mm^2^).

## Abbreviations

AD: axial diffusivity
ADI-R: Autism Diagnostic Interview-Revised
ADOS-G: Autism Diagnostic Observation Schedule-Generic
ASD: autism spectrum disorder
DQ: development quotient
DTI: diffusion tensor imaging
FA: fractional anisotropy
MD: mean diffusivity
MSEL: Mullen Scales of Early Learning
RD: radial Diffusivity
TCV: total cerebral volume
TD: typical development
