# Erratum: Sex differences in the corpus callosum in preschool-aged children with autism spectrum disorder

**DOI:** 10.1186/s13229-015-0030-3

**Published:** 2015-06-20

**Authors:** Christine Wu Nordahl, Ana-Maria Iosif, Gregory S Young, Lee Michael Perry, Robert Dougherty, Aaron Lee, Deana Li, Michael H Buonocore, Tony Simon, Sally Rogers, Brian Wandell, David G Amaral

**Affiliations:** The MIND Institute, School of Medicine, University of California at Davis, 2805 50th Street, Sacramento, 95817 California USA; Department of Psychiatry and Behavioral Sciences, School of Medicine, University of California at Davis, Sacramento, 95817 California USA; Department of Public Health Sciences, School of Medicine, University of California at Davis, Davis, 95616 California USA; Department of Psychology, Stanford University, Stanford, 94305 California USA; Center for Cognitive and Neurobiological Imaging, Stanford University, Stanford, 94305 California USA; Department of Radiology, School of Medicine, University of California at Davis, Sacramento, 95817 California USA

## Erratum

Due to a publisher error, this article [1] was originally published with figures one and two interchanged. This error has now been corrected.
