# Supplementary material for: Sex differences in the corpus callosum in preschool-aged children with autism spectrum disorder
Source: Mol Autism. 2015 May 13;6:26. doi: 10.1186/s13229-015-0005-4 (PMC4429319; doi:10.1186/s13229-015-0005-4)
Supplement: Additional file 1: Table S1. — Assessment of head motion in diffusion-weighted images. Raw counts and percent of the participants with excluded volumes. [file 13229_2015_5_MOESM1_ESM.docx]

Supplemental Table 1. Assessment of head motion in diffusion-weighted images. Raw counts and percent of the participants with excluded volumes.

| # excluded volumes | ASD | | TD | |
| --- | --- | --- | --- | --- |
|  | Males | Females | Males | Females |
| 0 | 164 (79%) | 29 (66%) | 60 (64%) | 36 (69%) |
| 1 | 20 (9%) | 5 (11%) | 17 (18%) | 5 (9.6%) |
| 2 | 12 (5.8%) | 6 (13.6%) | 7 (7.4%) | 6 (11.5%) |
| 3 | 8 (3.9%) | 2 (4.5%) | 7 (7.4%) | 2 (3.8%) |
| 4 | 2 (1.0%) | 1 (2.3%) | 0 (0%) | 1 (1.9%) |
| 5 | 0 (0%) | 0 (0%) | 1 (1.1%) | 0 (0%) |
| 6 | 1 (0.5%) | 1 (2.3%) | 2 (2.1%) | 2 (3.8%) |
| Total | 207 | 44 | 94 | 52 |

Data are expressed as count (percent)
