# Supplementary material for: Sex differences in the corpus callosum in preschool-aged children with autism spectrum disorder
Source: Mol Autism. 2015 May 13;6:26. doi: 10.1186/s13229-015-0005-4 (PMC4429319; doi:10.1186/s13229-015-0005-4)
Supplement: Additional file 2: Table S2. — Summary (parameter estimates and standard errors) of the random-effect models assessing the relationship of diagnostic group, sex, age, and cortical projection zone subregions with midsagittal area. [file 13229_2015_5_MOESM2_ESM.docx]

**Supplemental Table 2.** Summary (parameter estimates and standard errors) of the random-effects models^1^ assessing the relationship of diagnostic group, sex, age,and cortical projection zone subregions with midsagittal area

| ***Model Variable*** | Estimate (*SE*) | *P-*value |
| --- | --- | --- |
| ***Effects on the TD group*** | | |
| Intercept | 69.86 (3.42) | <.0001 |
| Scanner Upgrade | 1.04 (1.05) | .32 |
| TCV^2^ | 5.73 (0.77) | <.0001 |
| Orbitofrontal | -20.22 (4.45) | <.0001 |
| Anterior Frontal | 89.72 (6.31) | <.0001 |
| Lateral Frontal | -14.96 (6.35) | 0.02 |
| Superior Frontal | 136.37 (5.82) | <.0001 |
| Superior Parietal | 17.34 (5.25) | .001 |
| Posterior Parietal | 35.31 (5.97) | <.0001 |
| Temporal | -10.73 (5.22) | .04 |
| Occipital | reference |  |
| Age (year) | 1.08 (0.83) | .19 |
| Age*Orbitofrontal | 0.03 (1.00) | .98 |
| Age *Anterior Frontal | 4.38 (1.88) | .02 |
| Age*Lateral Frontal | -3.56 (1.89) | .06 |
| Age*Superior Frontal | 7.28 (1.69) | <.0001 |
| Age*Superior Parietal | -5.27 (1.43) | .0003 |
| Age* Posterior Parietal | 1.45 (1.74) | .40 |
| Age*Temporal | -3.20 (1.38) | .02 |
| Age*Occipital | reference |  |
| ***Regional (Cortical Projection Zone Sub-regions) Differences for ASD females vs. TD females*** | | |
| ASD Diagnosis | -5.73 (4.80) | .23 |
| ASD*Orbitofrontal | 6.77 (6.33) | .29 |
| ASD*Anterior Frontal | 14.72 (8.90) | .10 |
| ASD*Lateral Frontal | 13.97 (9.04) | .12 |
| ASD*Superior Frontal | -11.76 (8.21) | .15 |
| ASD*Superior Parietal | -1.18 (7.44) | .87 |
| ASD*Posterior Parietal | -7.61 (8.39) | .37 |
| ASD*Temporal | 7.63 (7.38) | .30 |
| Occipital | reference |  |
| ***Regional (Cortical Projection Zone Sub-regions) Differences for TD males vs. TD females*** | | |
| Male Sex | -6.09 (4.16) | .14 |
| Male*Orbitofrontal | 5.46 (5.44) | .32 |
| Male*Anterior Frontal | -5.76 (7.59) | .45 |
| Male*Lateral Frontal | 6.28 (7.89) | .43 |
| Male*Superior Frontal | -2.08 (7.03) | .77 |
| Male*Superior Parietal | -3.01 (6.38) | .64 |
| Male*Posterior Parietal | 3.03 (7.19) | .67 |
| Male*Temporal | 6.36 (6.30) | .31 |
| Male*Occipital | reference |  |
| ***Regional (Cortical Projection Zone Subdivisions*ASD*Male Interaction*** | | |
| Male*ASD Diagnosis | 3.43 (5.67) | .55 |
| Male*ASD*Orbitofrontal | -11.45 (7.45) | .13 |
| Male*ASD*Anterior Frontal | 25.19 (10.45) | .02 |
| Male*ASD*Lateral Frontal | -14.85 (10.80) | .17 |
| Male*ASD*Superior Frontal | 6.13 (9.66) | .53 |
| Male*ASD*Superior Parietal | 0.56 (8.76) | .95 |
| Male*ASD*Posterior Parietal | 15.38 (9.87) | .12 |
| Male*ASD*Temporal | -9.39 (8.66) | .28 |
| Male*Occipital | reference |  |

^1^The reported models contain those two-way or three-way interactions that were significant when tested against relevant lower-order models.. Age and TCV were centered at Time 1 means, so the intercept can be interpreted as the average Occipital area for an average age and TCV typically developing (TD) female child, ^2^ Effects of TCV are reported for 100 mm^3^ increments.
