# Supplementary material for: Sex differences in the corpus callosum in preschool-aged children with autism spectrum disorder
Source: Mol Autism. 2015 May 13;6:26. doi: 10.1186/s13229-015-0005-4 (PMC4429319; doi:10.1186/s13229-015-0005-4)
Supplement: Additional file 3: Table S3. — Summary (parameter estimates and standard errors) of the random-effect models assessing the relationship of diagnostic group, sex, and age with diffusion parameters. [file 13229_2015_5_MOESM3_ESM.docx]

**Supplemental Table 3.** Summary (parameter estimates and standard errors) of the random-effects models assessing the relationship of diagnostic group, sex, and age with diffusion parameters

|  | **Fractional Anisotropy** | | | **Mean Diffusivity** | | **Radial Diffusivity** | | **Axial Diffusivity** | |
| --- | --- | --- | --- | --- | --- | --- | --- | --- | --- |
| ***Model Variable*** | Estimate (SE) | *P-*value | | Estimate (SE) | *P-*value | Estimate (SE) | *P-*value | Estimate (SE) | *P-*value |
| ***Effects on the TD group*** | | | |  |  |  |  |  |  |
| Intercept | 0.713 (0.006) | <.0001 | | 0.941 (0.011) | <.0001 | 0.470 (0.013) | <.0001 | 1.884 (0.014) | <.0001 |
| Male Sex | 0.010 (0.005) | .03 | | 0.009 (0.012) | .45 | 0.004 (0.014) | .76 | 0.019 (0.014) | .17 |
| Age (year) | -0.003 (0.002) | .15 | | -0.011 (0.004) | .004 | -0.008 (0.004) | .04 | -0.017 (0.005) | .002 |
| Scanner Upgrade Diagnosis | -0.089 (0.003) | <.0001 | | 0.108 (0.005) | <.0001 | 0.150 (0.005) | <.0001 | 0.025 (0.005) | <.0001 |
| ***Differences from the TD group*** | | | | | | | | | |
| ASD Diagnosis | -0.003 (0.004) | .47 | | 0.041 (0.014) | .003 | 0.040 (0.016) | .01 | 0.044 (0.016) | .006 |
| Male*ASD^1^ | - | - | | -0.044 (0.017) | .008 | -0.042 (0.019) | .03 | -0.050 (0.019 | .009 |
| ***Regional Differences from Occipital*** | | | |  |  |  |  |  |  |
| Orbitofrontal | -0.051 (0.006) | <.0001 | | -0.049 (0.008) | <.0001 | 0.013 (0.009) | .18 | -0.175 (0.011) | <.0001 |
| Anterior Frontal | -0.045 (0.004) | <.0001 | | 0.028 (0.007) | .0001 | 0.055 (0.008) | <.0001 | 0.026 (0.009) | .004 |
| Lateral Frontal | -0.103 (0.010) | <.0001 | | 0.161 (0.022) | <.0001 | 0.215 (0.025) | <.0001 | 0.055 (0.019) | .005 |
| Superior Frontal | -0.066 (0.004) | <.0001 | | 0.045 (0.008) | <.0001 | 0.090 (0.008) | <.0001 | -0.047 (0.009) | <.0001 |
| Superior Parietal | -0.161 (0.007) | <.0001 | | 0.134 (0.012) | <.0001 | 0.221 (0.013) | <.0001 | -0.040 (0.017) | .02 |
| Posterior Parietal | -0.066 (0.005) | <.0001 | | 0.021 (0.008) | .01 | 0.062 (0.009) | <.0001 | 0.061 (0.011) | <.0001 |
| Temporal | 0.034 (0.005) | <.0001 | | 0.013 (0.009) | .14 | -0.026 (0.010) | <.0001 | 0.091 (0.010) | <.0001 |
| Occipital | reference |  | |  |  |  |  |  |  |
| ***Regional Differences in yearly rate of growth from Occipital*** | | | | | | | | | |
| Orbitofrontal | 0.000 (0.004) | | <.95 | 0.006 (0.005) | .24 | 0.002 (0.006) | .80 | 0.016 (0.007) | .03 |
| Anterior Frontal | 0.010 (0.003) | | .0003 | -0.005 (0.005) | .24 | -0.014 (0.005) | .007 | 0.011 (0.006) | .06 |
| Lateral Frontal | 0.006 (0.007) | | .36 | -0.009 (0.016) | .56 | -0.015 (0.018) | .42 | 0.001 (0.014) | .92 |
| Superior Frontal | -0.006 (0.003) | | <.03 | 0.013 (0.005) | .006 | 0.010 (0.005) | .049 | 0.021 (0.006) | .001 |
| Superior Parietal | 0.024 (0.005) | | <.0001 | 0.011 (0.008) | .19 | -0.014 (0.009) | .11 | 0.059 (0.012) | <.0001 |
| Posterior Parietal | -0.005 (0.003) | | <.13 | 0.017 (0.006) | .003 | 0.014 (0.006) | .02 | 0.023 (0.007) | .002 |
| Temporal | -0.009 (0.003) | | .007 | 0.009 (0.006) | .13 | 0.015 (0.007) | .03 | -0.002 (0.007) | .78 |
| Occipital | reference | |  |  |  |  |  |  |  |

^1^ The interaction between diagnosis and sex was not significant (*p* =.08), so it was not retained in the final model
