# Supplementary material for: Sex differences in the corpus callosum in preschool-aged children with autism spectrum disorder
Source: Mol Autism. 2015 May 13;6:26. doi: 10.1186/s13229-015-0005-4 (PMC4429319; doi:10.1186/s13229-015-0005-4)
Supplement: Additional file 4: Table S4. — Summary (parameter estimates and standard errors) of the random-effect model assessing the relationship of diagnostic group, sex, age, and Witelson subdivision with corpus callosum area (mm2). [file 13229_2015_5_MOESM4_ESM.docx]

**Supplemental Table 4.** Summary (parameter estimates and standard errors) of the random-effects model^1^ assessing the relationship of diagnostic group, sex,age, and Witleson subdivision with corpus callosum area (mm^2^)

| ***Model Variable*** | Estimate (SE) | *P-*value |
| --- | --- | --- |
| ***Effects on the TD group*** | | |
| Intercept | 142.42 (1.57) | <.0001 |
| TCV | 2.92 (0.44) | <.0001 |
| Age (years) | 3.60 (0.79) | <.0001 |
| Male Sex | -0.49 (1.24) | .69 |
| ***Differences from the TD group*** | | |
| ASD Diagnosis | -2.14 (1.07) | .047 |
| ***Regional Differences from Splenium*** | | |
| Rostrum | -124.19 (1.22) | <.0001 |
| Genu | -32.42 (1.48) | <.0001 |
| Rostral Body | -64.87 (1.25) | <.0001 |
| Anterior midbody | -82.26 (1.23) | <.0001 |
| Posterior midbody | -90.11 (1.23) | <.0001 |
| Isthmus | -100.29 (1.22) | <.0001 |
| Splenium | reference |  |
| ***Regional Differences from Splenium in yearly rate of change (age*region)*** | | |
| Rostrum | -4.66 (0.80) | <.0001 |
| Genu | 0.16 (1.04) | .88 |
| Rostral Body | -2.02 (0.83) | .02 |
| Anterior midbody | -2.10 (0.80) | .01 |
| Posterior midbody | -3.12 (0.81) | <.001 |
| Isthmus | -2.68 (0.80) | .001 |
| Splenium | reference |  |

^1^The reported models contain only significant interactions. We also included and tested terms for other two-way interactions, but none were significant and were not retained in the reported model. Age and TCV were centered at the means in the TD group at Time 1, so the intercept can be interpreted as the average corpus callosum area for an average age and average TCV in a TD child.
